# Partnerships for the Sustainable Development Goals: a call for more science

**DOI:** 10.3389/fnut.2024.1347593

**Published:** 2024-04-11

**Authors:** Elliot M. Berry, Barbara Burlingame, Johannes le Coutre

**Affiliations:** ^1^Braun School of Public Health, Hebrew University - Hadassah Medical School, Jerusalem, Israel; ^2^Riddet Institute, Massey University, Palmerston North, New Zealand; ^3^School of Chemical Engineering, University of New South Wales, Sydney, NSW, Australia; ^4^Australian Human Rights Institute, University of New South Wales, Sydney, NSW, Australia

**Keywords:** SDG 17, food security, partnerships for the goals, SDG 1, SDG 4, SDG 2, SDG 9

## 1 Introduction

The world is not on track for achieving the Sustainable Development Goals (SDGs) and the 2030 Agenda. The current geopolitical condition, impacted by the COVID-19 pandemic, wars in Ukraine and the Middle East, and the ever-increasing effects of climate change, has led to a global polyfactorial crisis, revealing a world ill-equipped to cope and improve. The context of global development is difficult to explain in hindsight, and it has become impossible to develop trustworthy forecasting for all sectors of society, government, and the environment.

Moreover, there is a growing consensus about the pivotal role of current food systems at the center of this crisis. Global food systems are not only the culprit behind broad aspects of environmental damage, but currently, we are also seeing their vulnerability and frailty in light of climate change.

We believe this grave situation can be leveraged to produce positive outcomes (1, 2) and the design of circular food systems aimed toward achieving the Sustainable Development Goals at both national and international levels.

However, for this to occur, a major re-thinking is required of the interface among primary, secondary, and tertiary sectors, and most pressingly the multidisciplinary activities of food systems. We discuss these challenges and suggest some of the approaches necessary to make this happen. At the core of our suggestion will be a call for partnerships for more open science, education, and capacity building as stated in Sustainable Development Goal 17, i.e., *PARTNERSHIPS FOR THE GOALS* (strengthen the means of implementation and revitalize the global partnership for sustainable development).

### 1.1 Values in the pursuit of knowledge

All SDGs are involved in every aspect of food security. When reflecting on the original four dimensions of food security (access, availability, utilization, and stability), it became increasingly clear that for food security *per se*, and for achieving the SDGs, at least two additional elements were needed: sustainability and agency (3, 4). Sustainability (in the context of environment and ecosystems) is the overarching and obvious feature, while agency brings power to people and communities, and a seat at the table for partnerships.

The SDGs convey the necessity for incorporating diverse knowledge systems to meet the targets. Different views have been expressed about the value of describing societies in terms of transitioning from an agrarian to an industrial society and, finally, to a knowledge-based society (5). However, consensus seems to exist on the general need for knowledge to initiate societal changes.

Genuinely, the pursuit of knowledge is enhanced through partnerships and driven by more/better science and shared data (Target 17.16), education and knowledge sharing (Target 17.6), and capacity building (Targets 17.7 and 17.8). These activities are directly and indirectly contained in all the SDGs but are specifically elaborated through two goals, i.e., SDG 4 and SDG 9.

SDG 4 is defined as *QUALITY EDUCATION* with the aim of ensuring inclusive and equitable opportunities for education in science, arts, and humanities and promoting lifelong learning prospects for all, and SDG 9 is defined as *INDUSTRY, INNOVATION, AND INFRASTRUCTURE* with the aim of building resilient infrastructure and promoting sustainable industrialization and foster innovation. By extension, both SDG 4 and SDG 9 also guide the conduct of science, technology, engineering, and mathematics (STEM) and capacity building.

With abundant discussion of SDG interdependencies in the literature, it is reasonable to just assert here that both SDG 4 and SDG 9 are critical for meeting all objectives linked with SDG 17 (6–8).

### 1.2 Science as an agent of change

Historically, the development of human society, the setting of regional, national, and global priorities, and the decision-making for future developments have often been driven by commerce or ideology. In times of crisis, the bandwidth for wrong decisions is narrow, systems are less resilient, and mistakes are less well tolerated and have more serious consequences. This vulnerability is enhanced when it is not only one crisis but, instead, multiple parallel crises.

Ever since the Age of Enlightenment in the 17^th^ and 18^th^ centuries, the pursuit of progress through conclusions based on evidence-based thinking has been the predominant method of building the future in many European countries and subsequently in most parts of the world.

In an unprecedented way, during the global COVID-19 pandemic from 2020 to 2022, we have seen the extraordinary power of scientific research, applied scientific development, and international cooperation when there is a sense of urgency. Following the publication of the coronavirus sequence, it took Özlem Türeci and Uğur Şahin, the two founders of BioNTech in Germany, less than a weekend to devise a meaningful strategy for the development of an effective COVID-19 vaccine. In hindsight, we know this vaccine saved millions of human lives. The scientific method, characterized by its evidence-based and logical approach to problem-solving, continues to be a crucial tool for effectively addressing our current and future global challenges. The scientific approach is transparent and free of hidden agendas, ideologies, or other value systems that do not directly contribute to finding solutions.

What is the state of 21^st^ century science and what kind of scientific practice is needed to successfully tackle our current poly crisis predicament?

A recent paper published in “Nature” makes the powerful observation that science has become predominantly an incremental pursuit of discovery, with few disruptive breakthroughs being conducted (9). If this is so, then, perhaps, we might be performing science in an untargeted (and inefficient) way. Clearly, however, science is essential as problems and crises are more than abundant. It might be helpful to broaden the scope, definition, and content of scientific thinking toward a more holistic, interdisciplinary activity with new frameworks. One such framework could be the use of complex adaptive systems (CAS) as a target for problem-solving (10, 11). The CAS concept reflects the complexity, non-linear dynamics, and unpredictability of multiple naturally occurring systems, such as climate, animal colonies, or developing biological systems. Our global crises, i.e., climate change, food insecurity, and loss of biodiversity, are governed by the CAS principles, and political parties and societal stakeholders to a specific problem can also be seen as CAS.

Another, already more established framework is given through the concept of *industry 4.0*, where value streams, novel technologies, and sustainability metrics are equal pillars for innovation to be implemented (12–14).

Scientific breakthroughs are dependent on creative thinking, policy enablers, and often very large budgets. In our world, policy development does reflect a scientific discipline as well. For example, when considering sustainable diets and food systems, such policy enablers include actors involved in all sectors of society.

## 2 Discussion

### 2.1 Science plays a central role in achieving all Sustainable Development Goals

The three core metrics public health (15), planetary health (i.e., sustainability) (16), and economic health interact globally, potentially leading to either a positive feedback loop or a negative downward spiral in achieving all SDGs.

Central to this triad appear our global food systems (17) with an ambiguous position in that they are the key culprits behind the poly crises, and yet, at the same time, it is only through their reforms that we can mitigate them. In other words, they are both part of the problems and their solutions.

It is not only about the availability of food; it is also crucial to ensure that communities in Least Developed Countries have the means to generate incomes and develop purchasing power. Social inequalities and poverty are major determinants of food insecurity, health, and malnutrition among many vulnerable sectors of the population, which is reflected by SDG 1 and SDG 10. Tragically, approximately two billion people worldwide suffer from micronutrient deficiency (hidden hunger), obesity, moderate or severe food insecurity, or a combination of these issues. The pandemic of obesity is causing far greater economic and health issues than that of COVID-19 (18). Undernutrition is a major contributor to morbidity and mortality from *communicable* diseases such as HIV/AIDS, TB, malaria, and diarrheal disease, while overnutrition/obesity leads to the major *non-communicable* diseases such as diabetes and diabesity, cancer, cardiovascular, and joint diseases with their attendant social disadvantages.

People and communities suffering from food insecurity are often among the most exposed to the effects of climate change, which threatens approximately one-third of the world's food production. However, at the same time, our food systems, especially food procurement and agriculture, are among the main culprits responsible for climate breakdown. With climate change accelerating and an increase in droughts and floods around the world, global food systems will be at risk of being severely compromised. In addition, some 30% of all food produced for human consumption is lost or wasted. If this were prevented, then there would be enough food to feed the planet, but this is not yet achievable because of geopolitical and economic considerations overriding those of equity. The transformation of food systems that is needed to address many of these issues requires profound leadership to induce behavioral changes (19, 20).

While evidence-based policies and programs have dominated science partnerships, diverse knowledge systems have been increasingly recognized as essential for achieving the SDGs. Indigenous Peoples' traditional knowledge systems continue to gain recognition, respect, and value from the scientific and academic communities. Indigenous Peoples' knowledge has methodological, substantive, and contextual strengths equal to or indeed beyond those of many (dominant) scientific study designs. Backed by centuries, if not millennia, of systematic observations, experiences, trials, and practice, traditional knowledge holds much relevance to contemporary food systems challenges. With the imperative of greater “agency” in SDG processes, and partnerships in particular, Indigenous Peoples involved in the co-creation of knowledge are contributing to science-based actions for addressing all the goals and targets. The recognition that there is no contradiction between innovation and tradition, and that lived science is as legitimate as empirical sciences, is gaining wider acceptance (21, 22).

### 2.2 Enduring issues

The Committee on World Food Security (CFS) has requested that the High-Level Panel of Experts (HLPE) update be undertaken at least every 4 years on critical and emerging issues. However, in 2022, the HLPE independently renamed the update to include enduring issues, recognizing that some of the key problems previously identified as affecting food security and nutrition, and as presenting barriers to achieving the SDGs, are still very pressing and therefore deserve continuing attention and resources (3, 23). At the special session of the CFS in October 2022, the Special Rapporteur on the Right to Food remarked that even if the Ukraine war ended now, and COVID-19 had never existed, food insecurity and malnutrition in all its forms were critical and enduring problems that deserved equal attention.

**Figure 1:**
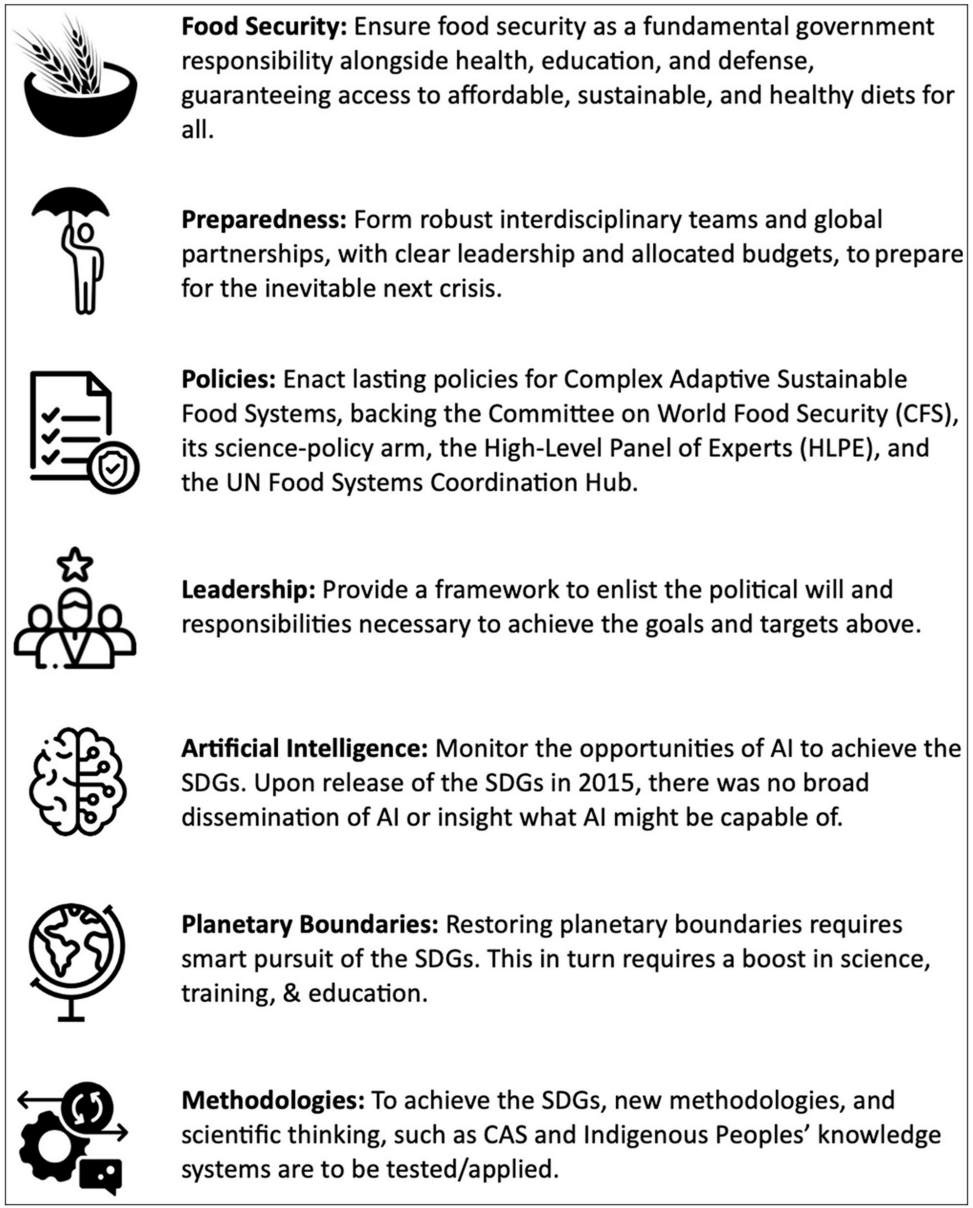
Framework of action points for achieving the Sustainable Development Goals. For more information on planetary boundaries see (24, 25).

From the Universal Declaration of Human Rights in 1948 to the resolutions of the UN Food System Summit in 2021, and the Committee on World Food Security through the present day, there is a plethora of resolutions and recommendations with signatories from most countries that languish without the political actions promised and with no accountability for their failure to act. With a view on the global next steps and the interplay between technology and policy, a framework of action points is presented in Figure 1, focusing on high-level feasibility and implementation. All action points can be directly implemented, and they speak for themselves. While not discussed explicitly, it is important to include strategic metrics and opportunities, such as artificial intelligence, preparedness, and planetary boundaries into this framework. We do not need more recommendations. We need action.

## Author contributions

BB: Writing – review & editing, Writing – original draft, Validation, Methodology, Investigation, Conceptualization. EB: Writing – review & editing, Writing – original draft, Validation, Methodology, Investigation, Conceptualization. JC: Writing – review & editing, Writing – original draft, Validation, Resources, Methodology, Investigation, Funding acquisition, Conceptualization.
